# The study of the relationship between moderate to severe sleep obstructive apnea and cognitive impairment, anxiety, and depression

**DOI:** 10.3389/fneur.2024.1363005

**Published:** 2024-05-10

**Authors:** Yanan Hong, Chong Pei, Lingli Hao, Kang Xu, Feifei Liu, Zhen Ding

**Affiliations:** Department of Respiratory and Critical Care Medicine, The Third Affiliated Hospital of Anhui Medical University (The First People’s Hospital of Hefei), Hefei, Anhui, China

**Keywords:** OSA, Apnea-Hypopnea Index, polysomnography, cognitive function, depression, anxiety

## Abstract

**Objective:**

The present study endeavored to investigate the interconnection between obstructive sleep apnea (OSA) and cognitive function, alongside the manifestations of depression and anxiety. Simultaneously, an analysis was conducted to discern the factors exerting influence upon cognitive function.

**Methods:**

A cohort of 102 patients, who had undergone polysomnography (PSG) at Binhu Hospital, Anhui Medical University, between January 2022 and June 2023, was encompassed in the study. Employing the PSG findings, these individuals were classified into two distinct categories: the grouping consisted of those with either negligible or mild OSA, and the other comprised individuals with moderate to severe OSA. Utilizing the Montreal Cognitive Assessment (MoCA-Beijing), Stroop Color and Word Test (SCWT), Digit Span Test (DST), Self-rating Depression Scale (SDS), and Self-rating Anxiety Scale (SAS), scores were recorded and analysed for each of the respective assessments. Additionally, discrepancies and associations between these groups were also scrutinized.

**Results:**

The group exhibiting moderate to severe OSA demonstrated significantly elevated measurements in parameters such as neck circumference, BMI, completion times for SCWT-A, B, C, Sleep Inefficiency Index (SIE), SAS, and SDS, in comparison to the No or Mild OSA group. Furthermore, the moderate–severe OSA group manifested notably diminished MoCA scores in areas of visual–spatial and executive function, memory, language, abstraction, delayed recall, orientation, total MoCA score, lowest oxygen saturation (LSaO2), average oxygen saturation, Digit Span Test-backward(DST-b), and Digit Span Test-forward(DST-f), as contrasted with the no-mild OSA group. These inter-group disparities exhibited statistical significance (*p* < 0.05). The MoCA total score portrayed inverse correlations with age, Apnea-Hypopnea Index (AHI), BMI, SIE, SAS, SDS, CT90%, AHT90%, and Hypoxic Apnea Duration (HAD) (ranging from −0.380 to −0.481, *p* < 0.05). Conversely, it displayed positive correlations with DST-f, DST-b, LSaO2, and average oxygen saturation (ranging from 0.414 to 0.744, *p* < 0.05). Neck circumference, AHI, and SAS were autonomously linked to MoCA scores (OR = 1.401, 1.028, 1.070, *p* < 0.05), while AHI exhibited an independent correlation with SDS and SAS scores (OR = 1.001, *p* = 0.003).

**Conclusion:**

Patients grappling with moderate to severe OSA frequently reveal cognitive impairment and concomitant emotional predicaments encompassing depression and anxiety. These manifestations share an intimate association with AHI, LSaO2, and average oxygen saturation. Notably, anxiety, when coupled with OSA, emerges as an autonomous influential element impinging upon cognitive impairment.

## 1 Introduction

Obstructive Sleep apnea (OSA) stands as a prevalent respiratory-linked sleep disorder characterized by recurrent obstruction of the upper airway during slumber. This condition precipitates episodic nocturnal hypoxia and upheaval of sleep architecture. Clinical features encompass habitual snoring, sleep-associated apnoeic or hypopnoeic events, while diurnal symptoms encompass excessive daytime somnolence and fatigue (1). The profound impact of severe OSA on patients’ quality of life and its potential to give rise to diverse complications, spanning neurological, psychiatric, and cardiovascular domains, cannot be understated.

Recent years have witnessed investigations that proffer indications of OSA’s potential repercussions on cognitive function (2, 3), with particular emphasis on memory, executive function, processing speed, and attention (4). Such effects might be attributed to the recurrent nocturnal hypoxia experienced by OSA sufferers, instigating neuronal compromise within the hippocampus. Consequently, cognitive deficits and indelible memory and learning debilities transpire (5). In tandem, some scholars posit that OSA’s enduringly intermittent hypoxic episodes may instigate the breach of the blood–brain barrier (BBB) (6), the guardian of cerebral homeostasis. Such disruption could herald the release of beta-amyloid and Tau proteins, early harbinger biomarkers of cognitive dysfunction (7). This complex interplay signifies OSA’s plausible role in the genesis of cognitive impairment. Additionally, given the frequently concurrent presence of obesity among OSA patients, this comorbidity could potentially compound cognitive dysfunction. Notwithstanding, a conundrum persists concerning the causal link between OSA and cognitive deficits, compounded by a paucity of pertinent clinical substantiation.

Moreover, OSA patients grapple with persistent perturbations in sleep architecture, demonstrably linked with psychiatric ailments. Recurrent disruptions of rapid eye movement (REM) sleep, coupled with intermittent hypoxia and hypercapnia, exact a toll on the prefrontal cortex. This culminates in compromised behavioral, cognitive, and self-regulatory emotional capacities (8). Nonetheless, a definitive verdict regarding the causal nexus between OSA and emotional ailments such as depression and anxiety remains elusive. Moreover, the concurrent presence of somatic illnesses and emotional disturbances potentially amplifies the spectrum of cognitive dysfunction experienced by patients.

This study employed neuropsychological assessments and self-rating scales to examine the potential coexistence of cognitive deficits and emotional disturbances, such as depression and anxiety, among OSA patients. It scrutinized the intricate interplay between OSA and cognitive function, alongside depression and anxiety, aiming to pinpoint the precise facets of cognitive function susceptible to OSA’s influence. Furthermore, the study delineated the determinants influencing cognitive function and furnished clinical data to underpin research into the underlying mechanisms governing cognitive decline and emotional alterations within OSA patients.

## 2 Materials and methods

### 2.1 Study design and population

A total of 102 patients, scheduled for polysomnography at Anhui Medical University’s Binhu Hospital between January 2022 and June 2023, were encompassed in this investigation. Based on the polysomnography (PSG) findings, these participants were categorized into two groups: those exhibiting either negligible or mild OSA, and individuals with Moderate to Severe OSA.

The criteria for inclusion in the OSA group were as follows: (1) individuals were in the initial stages of an OSA diagnosis and had no previous history of respiratory device treatment; (2) participants displayed the capability to collaborate and fulfill both PSG and scale assessments; (3) clear mental acuity and unimpaired communication skills were evident; (4) informed consent for study participation was obtained.

The exclusion criteria were delineated as follows: (1) individuals with a medical history involving disorders affecting small airway resistance, such as chronic obstructive pulmonary disease (COPD) and bronchial asthma; (2) those with a history of cerebral infarction, cerebral hemorrhage, traumatic brain injury, or cranial trauma; (3) individuals afflicted by psychiatric ailments, neoplasms, or a history of alcohol or substance misuse; (4) participants grappling with primary emotional conditions or under the influence of central nervous system-affecting medications, including antidepressants or antipsychotics; (5) individuals diagnosed with or having a history of sleep disorders apart from OSA, such as narcolepsy; (6) those bearing confirmed diagnoses of conditions acknowledged to contribute to cognitive impairment, such as diabetes; (7) individuals lacking literacy, afflicted by color blindness, or affected by color vision deficiencies (9).

This study has been approved by Binhu District Committee of the Third Affiliated Hospital of Anhui Medical University (Ethics batch number: 2022-041).

### 2.2 Depression and anxiety assessment

To gage patients’ levels of depression and anxiety, the Self-rating Depression Scale (SDS) and Self-rating Anxiety Scale (SAS) were utilized. First introduced in 1964, the SDS encompasses physiological and psychological symptoms, primarily serving as a tool to screen for depressive symptoms. It offers an intuitive insight into patients’ subjective emotional states (9). The SAS, established in 1971, has found broad application in clinical contexts. It encompasses five emotional and 15 physical symptoms (10). Both scales are scored by summing values (raw scores) across 20 items and subsequently converting them into standard scores, using the formula standard score = raw score × 1.5. Scores falling below 50 are indicative of normal states, while those within the range of 50–60 suggest mild conditions. Scores ranging from 60 to 70 denote moderate states, and scores surpassing 70 indicate severe conditions.

### 2.3 Cognitive function tests

The Montreal Cognitive Assessment (MoCA) serves as a tool to appraise participants’ cognitive capabilities. Designed with a particular focus on Mild Cognitive Impairment (MCI), MoCA boasts high sensitivity and specificity. This assessment evaluates eight cognitive domains, encompassing visual–spatial and executive function, naming, memory, attention, language, abstraction, delayed recall, and orientation (11). The maximum achievable score is 30 points, with scores falling below 26 indicating the presence of cognitive impairment.

The Stroop Color and Word Test (SCWT) holds a prominent place in neuropsychological evaluations for both experimental and clinical purposes. Its central aim is to gage the capacity for inhibiting cognitive interference (12). Additionally, research has corroborated its efficacy in assessing other dimensions of cognitive function, including attention, processing speed, cognitive flexibility, and working memory (13). Comprising three cards, each adorned with 24 words, SCWT mandates the calculation of completion times for each card, alongside the computation of the Stroop Interference Effect (SIE), as derived from subtracting the completion time of Card C from that of Card B. Enhanced SIE values correspond to diminished interference inhibition efficiency.

The Digit Span Test (DST) primarily examines short-term memory and attention (14). It incorporates both the forward and backward Digit Span Tests, during which an examiner presents digits at a pace of one per second. Subsequently, the participant is tasked with accurately reproducing progressively lengthening digit sequences in both the forward (DST-f) and backward (DST-b) orders. The highest number of digits accurately recalled in sequence and in reverse formulates the forward and backward scores. Each level allows two attempts, and the examination concludes when a subject fails both endeavors.

### 2.4 Polysomnography

Polysomnography (PSG) has, since its inception in 1974, emerged as the quintessential sleep monitoring assessment and remains the gold standard for diagnosing OSA (15). PSG meticulously and continuously records a multitude of indicators throughout a full night’s sleep, encompassing electroencephalography, electrooculography, submental electromyography, thoracoabdominal movements, nasal airflow, oxygen saturation, and electrocardiography. This data is subject to automated analysis followed by manual verification on an item-by-item basis. To evaluate sleep apnea and hypopnoea, the Apnea-Hypopnea Index (AHI) is utilized, in compliance with the criteria set forth by the American Academy of Sleep Medicine. The monitoring procedure encompasses three principal components: (1) scrutiny of the subject’s sleep architecture (comprising sleep stages), sleep efficiency (assessed by the arousal index), and AHI; (2) assessment of the subject’s sleep-related breathing function, entailing the identification of apnoeic or hypopnoeic episodes, their typology (central, obstructive, or mixed), and severity; (3) evaluation of sleep-related cardiovascular function to ascertain any potential causative role or exacerbation of arrhythmias, hypertension, and related phenomena.

### 2.5 Statistical analysis

The analytical evaluation of data was carried out using SPSS 26.0 statistical software. To assess the normal distribution of continuous variables, the Kolmogorov–Smirnov test was employed. Normally distributed data were presented as mean ± standard deviation (x ± s), and between-group comparisons were accomplished using independent sample t-tests. Non-normally distributed data underwent comparison using the Mann–Whitney U test and were presented as median (interquartile range, IQR). Spearman correlation analysis was applied to explore the associations among MoCA total score, SAS and SDS scores, alongside other variables. Multiple logistic regression analysis was employed to discern the autonomous factors linked to the manifestation of cognitive impairment, depression, and anxiety. The threshold for statistical significance was set at *p* < 0.05.

## 3 Results

### 3.1 Comparison of cognitive function and mood between No or Mild OSA and moderate to severe OSA groups

Throughout the study duration, a total of 102 participants were enlisted. Based on the PSG outcomes, 37 individuals exhibiting an AHI < 15 events/h were designated as the No or Mild OSA group, whereas 65 subjects displaying an AHI ≥ 15 events/h were ascribed to the Moderate to Severe OSA group. The Moderate to Severe OSA group evinced notably higher measurements for neck circumference, BMI, SCWT-A, B, and C completion times, SIE, SAS, SDS scores. In contrast, this group demonstrated significantly reduced scores in MoCA domains including visuospatial and executive function, memory, language, abstraction, delayed recall, orientation, and overall MoCA score, LSaO2, mean oxygen saturation, DST-b, and DST-f, when juxtaposed against the No or Mild OSA group (*p* < 0.05) (refer to Table 1).

**Table 1:** Comparative analysis of cognitive function and mood: No or Mild OSA vs. moderate to severe OSA (statistical investigation).

Covariate	No or Mild OSA	Moderate to severe OSA	*p*
Age	35.30 ± 11.91	39.80 ± 9.03	0.051
Neck circumference	38.014 ± 3.90	40.36 ± 2.93	0.001^*^
BMI	25.62 (23.66, 28.00)	27.30 (25.98, 30.06)	0.001^*^
LSaO2	89.50 (86.25, 91.00)	70.00 (61.00, 80.00)	<0.001^**^
Average blood oxygen saturation	97.00 (96.35, 97.80)	95.00 (94.00, 96.05)	<0.001^**^
MoCA-Visual space and executive ability	5.00 (5.00, 5.00)	4.00 (4.00, 5.00)	<0.001^**^
MoCA-name	3.00 (3.00, 3.00)	3.00 (3.00, 3.00)	0.860
MoCA-memory	5.00 (5.00, 5.00)	4.00 (3.00, 4.50)	<0.001^**^
MoCA-attention	6.00 (6.00, 6.00)	6.00 (6.00, 6.00)	0.068
MoCA-expression	3.00 (3.00, 3.00)	3.00 (2.00, 3.00)	0.001^*^
MoCA-abstraction	2.00 (2.00, 2.00)	2.00 (1.00, 2.00)	0.007^*^
MoCA-Delayed recall	3.00 (2.00, 4.00)	2.00 (1.00, 2.00)	<0.001^**^
MoCA-orientation	6.00 (6.00, 6.00)	6.00 (6.00, 6.00)	0.033^*^
MoCA score	28.00 (27.00, 29.00)	25.00 (23.00, 27.00)	<0.001^**^
DST-f	10.00 (9.00, 11.00)	10.00 (8.00, 10.00)	0.014^*^
DST-b	7.00 (6.00, 8.00)	5.00 (4.00, 6.00)	<0.001^**^
SCWT-A Time consumed	15.12 (13.26, 16.91)	17.60 (14.58, 21.34)	<0.001^**^
SCWT-A Wrong quantity	0.00 (0.00, 0.00)	0.00 (0.00, 0.00)	0.085
SCWT-B Time consumed	18.16 (16.50, 19.87)	21.06 (16.78, 25.88)	0.001^*^
SCWT-B Wrong quantity	0.00 (0.00, 0.00)	0.00 (0.00, 0.00)	0.126
SCWT-C Time consumed	24.56 (21.13, 27.45)	32.00 (26.85, 41.05)	<0.001^**^
SCWT-C Wrong quantity	0.00 (0.00, 0.00)	0.00 (0.00, 0.00)	0.192
SIE	6.29 (4.10, 8.78)	10.82 (7.69, 13.44)	<0.001^**^
SAS	37.00 (34.00, 41.50)	43.00 (39.00, 50.00)	<0.001^**^
SDS	34.00 (33.00, 37.00)	49.00 (39.00, 64.50)	<0.001^**^

LSaO2, Lowest Oxygen Saturation; BMI, Body Mass Index; MoCA, Montreal Cognitive Assessment; DST-f, Forward Digit Span Test; DST-b, Backward Digit Span Test; SCWT, Stroop Color-Word Test; SIE, Stroop Interference Effect; SAS, Self-Rating Anxiety Scale; SDS, Self-Rating Depression Scale; *p < 0.05; **p < 0.001.

### 3.2 Comparison of clinical data between normal cognitive function group and cognitive impairment group

Employing a MoCA score < 26 as the benchmark, the cohort was bifurcated into two categories: the cognitive impairment group encompassing 59 subjects, and the normal cognitive function group comprising 43 subjects. Within the cognitive impairment group, distinctively higher values for age, neck circumference, BMI, AHI, SAS, SDS, CT90%, AHT90%, and HAD were noted, while concurrently demonstrating notably diminished lowest and mean oxygen saturation when juxtaposed against the normal cognitive function group (refer to Table 2). Spearman correlation analysis uncovered a negative correlation between the MoCA total score and age, AHI, BMI, SIE, SAS, SDS, CT90%, AHT90%, and HAD. Conversely, a positive correlation was established between the MoCA total score and DST-f, DST-b, LSaO2, and mean oxygen saturation (see Table 3).

**Table 2:** Comparative evaluation of clinical data: normal cognitive function group vs. abnormal cognitive function group (statistical analysis).

Covariate	Normal cognitive function group (*n* = 43)	Cognitive impairment group (*n* = 59)	*p*
Age	35.30 ± 10.65	39.98 ± 9.81	0.037^*^
Neck circumference	38.46 ± 3.33	40.28 ± 3.41	0.009^*^
BMI	26.30 (25.00, 28.37)	27.04 (25.40, 30.11)	0.085
LSaO2	88.00 (79.00, 90.00)	72.00 (66.00, 84.00)	<0.001^**^
Average blood oxygen saturation	97.00 (95.90, 97.00)	95.00 (94.00, 96.90)	0.003^*^
AHI	8.850 (2.70, 37.30)	55.40 (31.40, 77.20)	<0.001^**^
SAS	38.00 (35.00, 43.00)	43.00 (39.00, 51.00)	<0.001^**^
SDS	35.50 (33.00, 43.00)	48.00 (37.00, 64.00)	<0.001^**^
CT90%	0.30 (0.00, 4.90)	17.80 (1.50, 35.50)	<0.001^**^
AHT%	6.15 (2.50, 40.90)	50.80 (20.10, 61.10)	<0.001^**^
HAD (s)	222.50 (88.10, 1443.69)	1829.47 (724.80, 2200.80)	<0.001^**^
MAD (s)	24.73 (21.63, 27.40)	24.63 (22.13, 31.10)	0.432

AHI, Apnea-Hypopnea Index; CT90%, Percentage of time with oxygen saturation below 90% during total sleep time; AHT%, Percentage of time with apnea-hypopnea during total sleep time; MAD, Mean Apnea-Hypopnea Duration; HAD, Hourly Apnea-Hypopnea Duration; ^*^p < 0.05; ^**^p < 0.001.

The percentage of time with blood oxygen levels below 90% (CT90%) is calculated using the following formula: CT90% = (Time with blood oxygen levels below 90 min)/ (Total sleep time in minutes) × 100%.

The percentage of time with apnea-hypopnea events during sleep, known as Apnea-Hypopnea Time Percentage (AHT%), is calculated using the following formula: AHT% = [(Total duration of apnea events in minutes) + (Total duration of hypopnea events in minutes)]/(Total sleep time in minutes) × 100%.

The average apnea-hypopnea duration (MAD) (s) is calculated as follows: MAD = (Total apnea duration (s) + Total hypopnea duration (s))/ (Total apnea episodes + Total hypopnea episodes) × 100%.

The hourly apnea-hypopnea duration (HAD) (s) is derived by multiplying MAD by AHI.

**Table 3:** Correlation analysis of MoCA scores with adjacent indicators.

	*r*	*p*
Age	−0.380	<0.001^**^
Neck circumference	−0.064	0.528
AHI	−0.481	<0.001^**^
BMI	−0.227	0.022^*^
LSaO2	0.414	<0.001^**^
Average blood oxygen saturation	0.247	0.012^*^
DST-f	0.744	<0.001^**^
DST-b	0.562	<0.001^**^
SIE	−0.441	<0.001^**^
SAS	−0.387	<0.001^**^
SDS	−0.481	<0.001^**^
CT90%	−0.444	<0.001^**^
AHT%	−0.466	<0.001^**^
MAD (s)	−0.068	0.496
HAD (s)	−0.463	<0.001^**^

*p < 0.05; **p < 0.001.

### 3.3 Multiple logistic regression analysis of cognitive impairment in moderate/severe OSA patients

**Figure 1:**
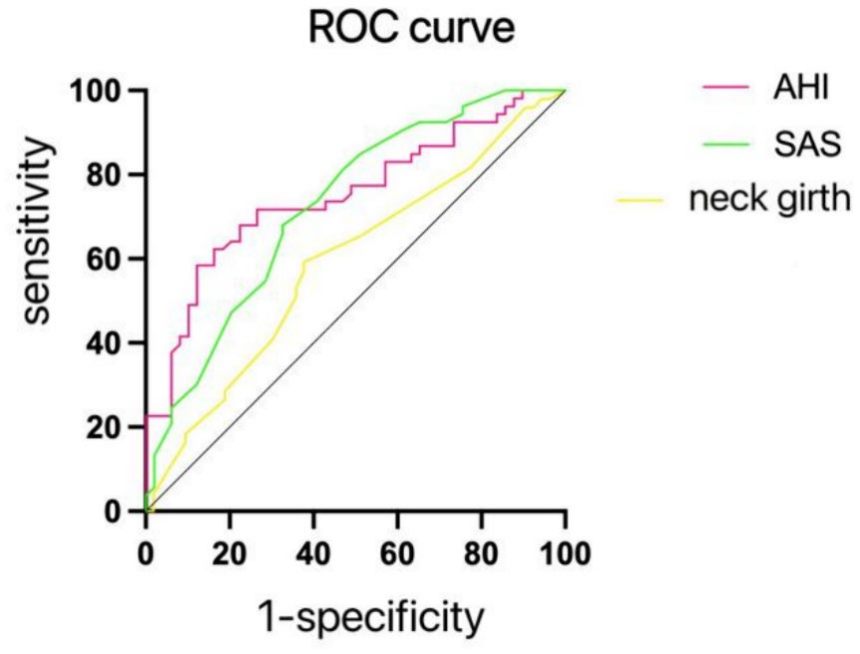
ROC curve for cognitive impairment.

The dependent variable in this context was cognitive impairment, with significant variables extracted from Table 2 being incorporated as independent variables within the regression model. Single-factor logistic regression analysis underscored significant associations across all variables. Upon their integration into multiple logistic regression analysis, neck circumference, AHI, and SAS emerged as autonomous risk factors influencing the prognosis of cognitive impairment in patients (see Table 4). Corresponding Receiver Operating Characteristic (ROC) curves were generated (refer to Figure 1 and Table 5).

**Table 4:** Multivariable logistic regression analysis: cognitive impairment in patients with OSA.

Variables	Univariate logistic regression analysis		Multivariate logistic regression analysis	
	OR(95%CI)	*p*	OR(95%CI)	*p*
Age	1.043 (1.002, 1.085)	0.040		
Neck circumference	1.172 (1.035, 1.327)	0.012	1.401 (1.702, 1.803)	0.014
LSaO2	0.952 (0.921, 0.985)	0.004		
Average blood oxygen saturation	0.706 (0.549, 0.909)	0.007		
AHI	1.033 (1.018, 1.049)	0.000	1.028 (1.007, 1.050)	0.010
SAS	1.123 (1.054, 1.200)	0.001	1.070 (0.991, 1.155)	0.083
SDS	1.073 (1.033, 1.115)	0.000		
CT90%	1.038 (1.012, 1.064)	0.003		
AHT%	1.038 (1.019, 1.057)	0.000		
HAD (s)	1.001 (1.001, 1.002)	0.000		

**Table 5:** AHI, SAS, neck circumference of the diagnosis of cognitive dysfunction related indicators.

	AUC	Sensitivity	Specificity	Threshold
AHI	0.785	0.897	0.714	15.60
SAS	0.718	0.638	0.714	41.50
Neck circumference	0.650	0.603	0.690	39.25

### 3.4 Comparison of clinical data between groups with and without depression and anxiety

Utilizing SAS and SDS scores, individuals securing scores surpassing 50 were classified into the abnormal group (comprising 36 subjects), while those garnering scores equalling or falling below 50 constituted the normal group (encompassing 66 subjects). An initial analysis of general data revealed that the abnormal group showcased significantly heightened AHI and CT90% readings in comparison to the normal group, with the abnormal group demonstrating a distinctly lower LSaO2 (refer to Table 6). Spearman correlation analysis illuminated a positive correlation between SAS and SDS scores, and variables such as AHI, SIE, CT90%, AHT, and HAD, while revealing a negative correlation with LSaO2, mean oxygen saturation, MoCA total score, DST-f, and DST-b (see Table 7).

**Table 6:** Comparative analysis of clinical data between the emotionally normal group and the depression and anxiety group (differential investigation).

	Normal	Depression and anxiety	*p*
Age	38.23 ± 10.39	37.95 ± 10.41	0.914
Neck circumference	39.50 (37.00, 42.50)	39.00 (37.00, 41.00)	0.333
BMI	26.80 (25.28,28.37)	27.00 (26.00, 30.80)	0.224
LSaO2	83.00 (71.00, 90.00)	68.00 (59.00, 82.00)	0.005^*^
Average blood oxygen saturation	96.00 (95.00, 97.00)	96.00 (95.00, 97.00)	0.512
AHI	27.80 (6.60, 66.40)	61.20 (42.00, 87.50)	0.018^*^
MoCA score	27.00 (24.00, 28.00)	25.00 (23.00, 27.00)	0.083
DST-f	10.00 (9.00, 10.00)	10.00 (8.00, 10.00)	0.676
DST-b	6.00 (4.00,7.00)	6.00 (4.00,7.00)	0.250
SIE	8.75 (5.73, 12.27)	9.04 (4.12, 11.96)	0.929
CT90%	1.50 (0.03, 22.10)	13.31 (1.38, 42.59)	0.036^*^
AHT%	15.99 (5.40, 60.20)	47.94 (27.60, 61.65)	0.075
HAD (s)	576.84 (193.80, 2168.40)	1726.12 (994.47, 2220.52)	0.074
MAD (s)	25.30 (21.66, 30.50)	24.21 (22.13, 31.09)	0.623

*p < 0.05.

**Table 7:** Correlation analysis of SAS and SDS scores with correlated indicators.

	*r*	*p*
Age	−0.380	<0.001**
Neck circumference	−0.064	0.528
AHI	−0.481	<0.001**
BMI	−0.227	0.022*
LSaO2	0.414	<0.001**
Average blood oxygen saturation	0.247	0.012*
DST-f	0.744	<0.001**
DST-b	0.562	<0.001**
SIE	−0.441	<0.001**
SAS	−0.387	<0.001**
SDS	−0.481	<0.001**
CT90%	−0.444	<0.001**
AHT%	−0.466	<0.001**
MAD (s)	−0.068	0.496
HAD (s)	−0.463	<0.001**

*Means *p* < 0.05, **Means *p* < 0.001.

### 3.5 Multiple logistic regression analysis of depression and anxiety in moderate to severe OSA patients

**Figure 2:**
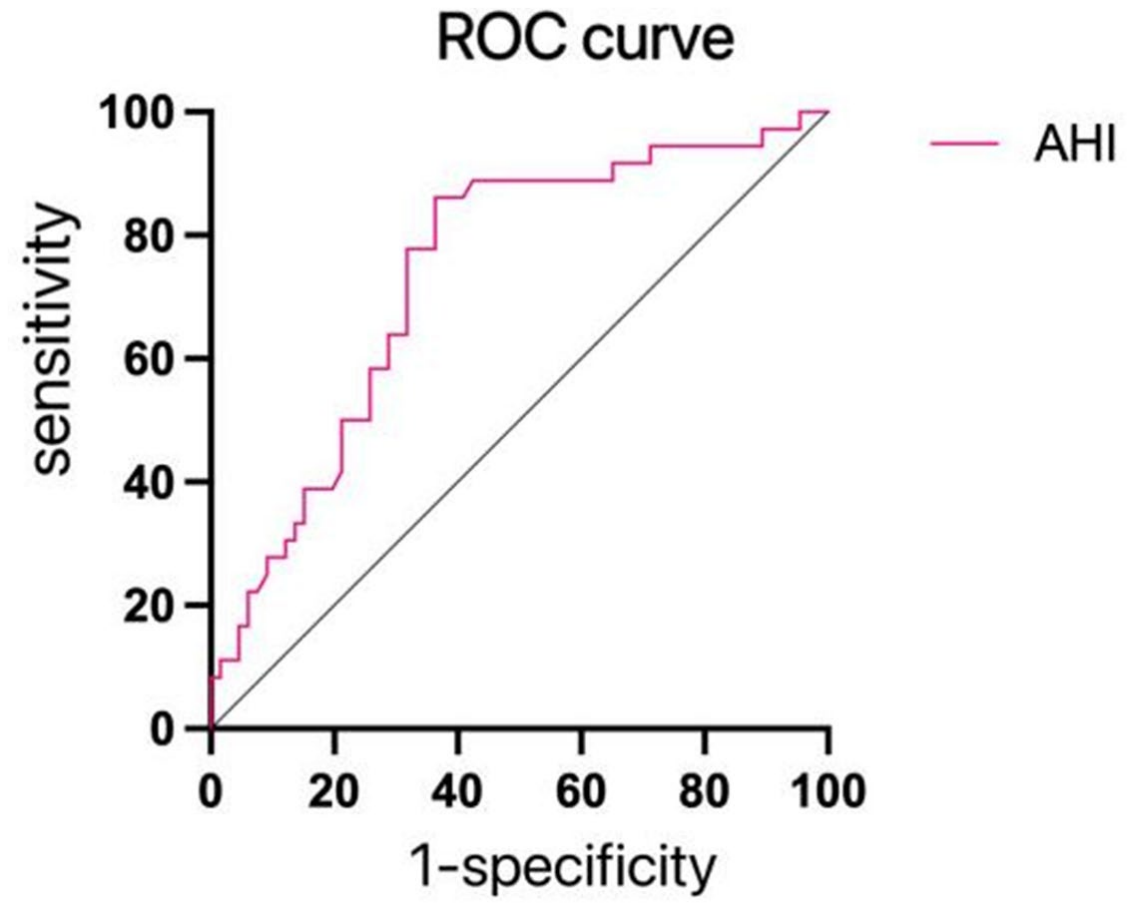
ROC curves for depression and anxiety. The AHI predicted the AUC of depression and anxiety was 0.737, the best cut-off value was 41.60 times/h, and the sensitivity and specificity were 77.3 and 60.0%, respectively, as shown in this figure.

With the presence of depression or anxiety-emergent emotional issues serving as the dependent variable, and notable variables from Table 6 forming the independent variables for regression, single-factor regression analysis indicated the statistical significance of AHI and LSaO2. These pivotal variables were subsequently integrated into a multiple logistic regression analysis, unveiling AHI as an independent risk factor for the occurrence of depression or anxiety-linked emotional complications in patients (refer to Table 8). The pertinent ROC curves were concurrently generated (see Figure 2).

**Table 8:** Multivariable logistic regression analysis: incidence of depression and anxiety in patients with OSA.

Variables	Univariate logistic regression analysis		Multivariate logistic regression analysis	
	OR(95%CI)	*p*	OR(95%CI)	*p*
AHI	1.018 (1.004, 1.032)	0.014	1.001 (0.979, 1.025)	0.003
LSaO2	0.953 (0.923, 0.984)	0.003		
CT90%	1.018 (0.998, 1.039)	0.083		

## 4 Discussion

The study findings are indicative of noteworthy disparities in cognitive function, depression, and anxiety-associated emotional challenges between the No or Mild OSA and the Moderate to Severe OSA groups. This discernment posits a deleterious impact of OSA on both cognitive function and emotional states, with this influence exhibiting escalated prominence in proportion to OSA severity (*AUC* = 0.785, 0.737). Substantiation through multiple logistic regression and correlation analyses serves to underscore that age, AHI, nocturnal oxygen saturation levels, and anxiety primarily intertwine with cognitive impairment. Concurrently, depression and anxiety align most prominently with AHI. Furthermore, it is worth noting that the existence of anxiety within the context of OSA stands as an autonomous factor contributing to the manifestation of cognitive impairment.

Cognitive function entails the intricate process through which the human brain acquires external stimuli, processes them, and internalizes them as mental activities, facilitating the assimilation and application of knowledge. It encompasses diverse facets such as memory, executive function, linguistic expression, spatial perception, numerical acumen, as well as comprehension and discernment (16). The meticulous analysis of clinical data in this investigation has underscored that OSA predominantly impacts cognitive domains associated with spatial perception, executive function, language, abstraction, delayed recall, orientation, the capacity to suppress cognitive interference, short-term memory, and attention. Similar studies, such as the one conducted by Andrew et al. (17), involving 1,084 participants subjected to PSG, MoCA, Rey Auditory Verbal Learning Test (RAVLT), and WAIS-IV Digit-Symbol Coding (DSC) to evaluate information processing speed, have demonstrated significantly diminished memory and information processing speed in OSA-afflicted individuals compared to those unaffected (*p* < 0.05). In the study conducted by Alexa et al. (18), it was discerned that adolescents grappling with both obesity and OSA displayed notably diminished executive function in comparison to the control group (*p* < 0.003). A staggering 45% of patients manifested compromised executive function, with a substantial 30% encountering clinically notable impairment. In the study orchestrated by Li et al. (19), involving 203 participants, attention and short-term memory assessments encompassing the Trail Making Test (TMT), DST, and Complex Figure Test (CFT) were administered. Outcomes demonstrated markedly reduced overall cognitive function in severe OSA patients, particularly in the realm of attention, in contrast to the non-OSA cohort (*p* < 0.05), thereby congruent with the present study’s discoveries. Nevertheless, in Yaouhi’s study (20), centered on a cohort of moderate to severe OSA patients, evaluations focusing on alertness, vigilance, divided attention, working memory, and episodic memory disclosed only mild memory and motor impairments, with no discernible cognitive deficits in other domains. Although the extent of cognitive debilitation precipitated by OSA remains a subject of contention, a preponderance of research substantiates the culmination that has been reached. Our study augments this body of knowledge by furnishing additional clinical substantiation to support this prevailing consensus.

Depression and anxiety are frequently encountered emotional challenges. Our experimental outcomes markedly indicate a considerably heightened incidence of depression and anxiety among patients afflicted with moderate to severe OSA, when compared to the control group (21, 22). This concurs harmoniously with prior meta-analyses, reinforcing the robustness of our findings. The corpus of our research furnishes pivotal clinical validation for this phenomenon. Our meticulous data scrutiny, encompassing correlation analysis and multiple logistic regression, has unveiled a direct, linear correlation between the frequency of apneas or hypopneas during nocturnal hours, quantified as AHI, and the severity of depression and anxiety. In succinct terms, a patient’s proclivity for frequent occurrences of these respiratory events during sleep correlates with the intensity of depression and anxiety experienced. AHI emerges as an independent risk factor underpinning the manifestation of depression and anxiety. The intricate mechanics underlying the emotional ramifications of OSA remain shrouded in uncertainty, yet multiple plausible theories have been posited. Primarily, OSA patients are often beleaguered by daytime fatigue and sleep disturbances engendered by the erratic nocturnal hypoxia and sleep architecture disruptions, significantly impinging on diurnal activities (23). Notably, Jackson, M. L.’s research revealed that a considerable 22.7% of 109 untreated OSA patients exhibited clinical depression, with 24.8% resorting to antidepressants (24). Significantly lower sleep quality and diminished quality of life were correlated with clinical depression. In a separate study, Carneiro-Barrera, A.’s findings accentuated the substantial enhancements achievable through weight loss and lifestyle interventions in mitigating OSA-induced daily functional impairment and psychological manifestations (25). Secondly, OSA patients routinely manifest neurochemical imbalances (26). Research conducted by Vgontzas, (27) spotlighted markedly higher plasma concentrations of inflammatory and fatigue-inducing cytokines, such as tumor necrosis factor-α and interleukin-6, among obese male OSA patients compared to their non-OSA obese counterparts. These cytokines have been acknowledged as contributory factors to daytime sleepiness. The application of etanercept, a drug neutralizing tumor necrosis factor-α and inflammatory cytokines (28), in experimental settings on OSA-afflicted obese males led to a significant attenuation of daytime sleepiness. This intervention reaffirmed the profound interrelation between cytokine elevation and OSA (29). Lastly, the nexus between chronic diseases and depression is firmly established (30). OSA patients are often confronted with an array of chronic complications, encompassing obesity, hypertension, cardiovascular afflictions, and diabetes. Emotional struggles, typified by depression and anxiety, might transpire as secondary ramifications in the wake of these chronic maladies.

Without a doubt, OSA is significantly associated with cognitive functions and depression, anxiety, which bears important implications for the diagnosis and treatment of OSA. Firstly, from a diagnostic perspective, understanding that OSA can lead to cognitive decline and emotional issues helps us diagnose the patient’s condition more accurately. If a patient exhibits symptoms such as lack of concentration, memory loss, decision-making difficulties, or emotional problems (such as irritability, low mood, etc.), doctors might consider OSA as a potential cause. Secondly, regarding treatment, this conclusion underscores the importance of early diagnosis and timely treatment. Since OSA can cause cognitive decline and emotional issues, diagnosing and treating it promptly can prevent or at least slow the emergence of these negative effects. Moreover, treatment approaches need to consider the patient’s cognitive and emotional status. For instance, patients already experiencing cognitive decline and emotional issues might require a more comprehensive treatment strategy, including medication, psychotherapy, and lifestyle adjustments. Comparing cognitive functions and emotional changes before and after treatment can also evaluate the treatment’s effectiveness, allowing adjustments to the treatment plan as needed.

Furthermore, our study tentatively ascertained that the coexistence of anxiety and OSA serves as an autonomous catalyst for cognitive impairment, signifying that the amalgamation of anxiety with OSA might potentially exacerbate cognitive decline. Nevertheless, the dearth of pertinent mechanistic investigations and corresponding clinical trials underscores the necessity for further probing and exploration.

Continuous Positive Airway Pressure (CPAP) stands as a potent therapeutic modality for OSA management. Its principal mechanism revolves around augmenting positive pressure within the pharyngeal cavity to counterbalance the negative pressure incurred during inhalation, thus averting airway collapse. The extant body of research, fortified by meticulous meta-analyses, postulates that CPAP intervention not only ameliorates daytime sleepiness and emotional perturbations within OSA patients, but also exerts a discernible impact on enhancing cognitive function (31). Assorted clinical studies have corroborated the salutary effects of CPAP treatment (32–34), spotlighting marked enhancements in attention, working memory, and executive function post-treatment. Concurrently, symptoms of depression and anxiety are markedly mitigated. Recent investigations have proffered the notion that certain pharmacological agents hold the potential to alleviate clinical manifestations of OSA. Murillo-Rodríguez, E., for instance, validated the efficacy of modafinil in significantly ameliorating daytime hypersomnia resulting from OSA, concomitantly abating symptoms of depression and cognitive decline (35).

It is imperative to acknowledge certain limitations inherent in our study. Primarily, the relatively modest sample size warrants consideration for future research, with a recommendation to expand the sample pool to substantiate the intricate interplay between OSA, cognitive function, and emotional dynamics. Additionally, the absence of a post-treatment follow-up for individuals grappling with moderate to severe OSA imparts a sense of incompleteness to our investigation, warranting further comprehensive exploration in subsequent studies.

Moderate to severe OSA is demonstrably associated with cognitive impairment as well as the manifestation of depression and anxiety. These neurologic and psychological alterations are intimately linked to the AHI. In essence, the degree of OSA severity serves as a robust predictor, significantly anticipating the likelihood of cognitive impairment, depression, and anxiety. Timely identification and intervention of OSA also hold the potential to avert the emergence of these interconnected morbidities.

## Data availability statement

The raw data supporting the conclusions of this article will be made available by the authors, without undue reservation.

## Ethics statement

The studies involving human participants were reviewed and approved by the ethics committee of the Third Affiliated Hospital of Anhui Medical University (The First People’s Hospital of Hefei). The patients/participants provided their written informed consent to participate in this study.

## Author contributions

YH: Writing – original draft, Writing – review & editing. CP: Data curation, Writing – review & editing. LH: Data curation, Software, Writing – review & editing. KX: Data curation, Writing – review & editing. FL: Data curation, Software, Writing – review & editing. ZD: Conceptualization, Project administration, Funding acquisition, Writing – review & editing.
